# Agro-industrial byproducts in rabbit food: Case of the complex of detoxified apricot kernel cake and dehydrated tomato pulp

**DOI:** 10.14202/vetworld.2021.744-750

**Published:** 2021-03-23

**Authors:** Yasmine Arbouche, Achour Mennani, Lamya Ouzzir, Rafik Arbouche, Fodil Arbouche

**Affiliations:** 1 Department of Agronomy, Faculty of Science of Nature and Life, University of Setif1, Algeria; 2 Department of Agronomy, Faculty of Science of Nature and Life, University of Ghardaia, Algeria

**Keywords:** agro-industrial byproducts, apricot kernel cake, rabbit fattening, tomato pulp, zootechnical performances

## Abstract

**Background and Aim:**

The use of agro-industrial byproducts as an unconventional source of raw materials for monogastric feed is one possible solution. This study aimed to determine the effects of incorporating detoxified apricot kernel meal (DAKM) as a substitute for soybean meal and dehydrated tomato pulp (DTP) as a substitute for alfalfa hay on the local rabbit fattening.

**Materials and Methods:**

A total of 120 white strain rabbits, weaned at 33 days, were randomly assigned to four groups of 30. The rabbits in each group were ringed, placed in cages at 6 rabbits/cage, and fed according to DAKM and DTP incorporation rates (0%, 30%, 40%, and 60%).

**Results:**

The weights at 77 days were improved (p<0.05) with unchanged mean daily intakes. The vast majority of slaughter parameters and carcass characteristics improved. The chemical composition of the meat constituents improved significantly, with a 60% increase in the protein content of the batch (26.55% vs. 28.53%), 38% reduction in the total feed cost, and 40 DA saved for each kilogram of feed consumed per rabbit. The relative economic efficiency improved in proportion to the substitution rates of soybean meal by DAKM and alfalfa hay by DTP.

**Conclusion:**

Substituting DAKM and DTP, as byproducts of agro-industrial processing, for up to 60% induced satisfactory results in rabbit fattening. Therefore, it would be more insightful to increase the incorporation rates to determine the optimal threshold.

## Introduction

Algeria is one of the top producers of rabbit meat in Africa. The intensive production of rabbits (*Oryctolagus cuniculus*) has several advantages that solve a part of the worldwide shortage in animal protein [1,2]. Rabbit diet accounts for up to 70% of production costs [3], which are mainly due to the importation of most raw materials used in feed formulas. The short-term solution lies in the introduction of agricultural and agro-industrial byproducts into feed formulas [4-8], which would make it possible, in the short term, to make meat products available to the poorest populations at a lower cost. In rabbit farming, agro-industrial byproducts, such as apricot kernel cake and date scraps, have been successfully introduced at a rate of 30% [9,10] and apricot kernel cake alone at a rate of 60% [11]. In the framework of the continuity of this approach, this study aimed to introduce the byproducts, such as the apricot kernel cake versus soybean cake and tomato pulp versus dehydrated alfalfa, in rabbit feed.

The tomato pulp generated by processing the latter is important in the northeast region of the country. This weeded crop is grown on an area of 21,434 ha for an estimated production of 1.235 million tons per year, 85% of which is concentrated in the Wilayas of Skikda, Annaba, Guelma, and El-Tarf [12]. With 27 processing units, about 13% of these are byproducts [13], or 160,602 T/year, consisting mainly of seeds, husks, and stalks.

The tomato pulp in rabbit feed can be effectively used for up to 20% incorporation rate, as it was tested extensively by some authors [14,15]. However, this rate varies as the chemical composition and nutritional value of this byproduct depend on the environment, the soil in particular, and the level of fertilization. The tomato pulp from national processing units is relatively rich in cellulosic fiber, protein, and fat (35.3%, 19.90%, and 16.1%, respectively) [15].

In Algeria, the stone fruit arboriculture is dominated by apricot trees, with about 46,000 hectares for an average annual production of 293,486 T, which is mainly concentrated (40%) in the region of Hodna, inducing the establishment of several processing units [12]. These units produce large quantities of byproducts each year, which are mostly cores. For El-Adawy *et al*. [16], the apricot kernel represents 15-16% of the apricot, while the almond represents 30-38% of the kernel. According to Ferradji *et al*. [17], the oil yield after crushing and pressing the apricot kernel is estimated to be 33% for 67% of the cake. Based on these data, the quantity of apricot kernel cake is estimated to be 106,970 T/year.

There are only a few studies on the use of apricot kernel cake as a protein source in animal feed, and its incorporation has been tested in broiler chicken [4], sheep in fertilizer [5], and in association with other byproducts in rabbit farming [9,10]. This coproduct contains a significant protein content (42.3%) [15].

## Materials and Methods

### Ethical approval

The present study was conducted after approval of the Institutional Animal Ethics Committee laboratory of the Agriculture department of Ghardaia University, Algeria.

### Study period and location

To guarantee the maintenance of environmental conditions, the test was carried out in a 200-m^2^ building with a pad cooling system and fans in a professional rabbit breeding center (Wilaya of Sétif, Algeria), during the month of April 2019.

### Animals, food, and experimental protocol

One hundred and twenty white strain bunnies, weaned at 33 days, were randomly divided into four groups of 30. The rabbits in each group were ringed and placed in cages at a rate of six rabbits per cage, that is, five repetitive batches per group. The sex of the rabbits was not given particular attention because until 15 [18] and 20 weeks of age [19], the males and females followed a similar growth curve and body composition.

The apricot kernel cake was supplied by an oil extraction unit located in Beni Ourtilane, Wilaya of Sétif. It was detoxified according to the method of Gabrial *et al*. [20]. The tomato pulp was supplied by the Nouvelle Ere canning factory in the industrial zone of the Wilaya of Sétif. It was dried in the sun for 3 days. The chemical compositions of detoxified apricot kernel meal (DAKM) and dehydrated tomato pulp (DTP) are shown in Table-1 [21,22].

**Table-1:** Chemical composition of detoxified almond cake (DAKM) and DTP in % DM.

Parameter	DAKM	DTP
Organic matter	96.7	95.12
Total nitrogenous matter	42.3	16.11
Crude fiber	7.7	37.92
Fat	10.4	10.22
Mineral content	3.3	4.88
Nitrogen-free extract	36.3	30.87
HCN (mg/100 g DM)	102	/
NDF	18.4	52.69
ADF	10.7	42.32
ADL	7.4	20.33
Hémicellulose	7.7	10.37
Gross energy (kcal/kg DM)	5180	4063
Digestible rabbit energy (kcal/kg DM)*	3984	2298
Digestible rabbit protein (g/kg of DM)*	336	130.6
Lysine (g/100 g of foodstuff)	1.8	1.0
Méthionine (g/100 g of foodstuff)	1.2	0.36
Cystine (g/100 g of foodstuff)	1.3	0.31

DM=Dry matter, NDF=Neutral detergent fiber, ADF=Acid detergent fiber, ADL=Acid detergent lignin, DAKM=Detoxified apricot kernel meal, DTP=Dehydrated tomato pulp.

* Estimated by the equation [21,22]

Four compound feeds were formulated using WUFFDA[23]; one control and three experimental feeds, in which we substituted 30, 40, and 60% of the soybean meal with DAKM and alfalfa hay with DTP (Table-2).

**Table-2:** Formula (kg/100 kg of feed) of feed distributed based on the substitution rate of soybean meal by DAKM and alfalfa by DTP.

Percentage of substitution	0%	30%	40%	60%
Corn	20	20	20	20
Soybean meal	12.7	8.9	7.6	5.1
Apricot kernel meal	0	3.8	5.1	7.6
Wheat bran	32	32	32	32
Wheat straw	4.7	4.7	4.7	4.7
Dried alfalfa	29	20.3	17.4	11.6
Tomato pulp	0	8.7	11.6	17.4
Salt (NaCl)	0.5	0.5	0.5	0.5
Rabbit premix (CMV)	0.5	0.5	0.5	0.5
Calcium carbonate	0.5	0.5	0.5	0.5
L-Lysine	0.08	0.08	0.08	0.08
DL-Methionine	0.02	0.02	0.02	0.02
Content of calculated nutrients				
Crude fiber (%)	15.7	15.1	15.8	16.4
NDF (%)	36.6	37.1	37.2	37.5
ADF (%)	20.1	20.5	20.6	20.9
ADL (%)	4.3	5.4	5.8	6.5
Hemicellulose (%)	16.6	16.6	16.6	16.6
Lysine (%)	0.83	0.82	0.85	0.88
Methionine (%)	0.29	0.27	0.26	0.24
Total sulfur amino acids (%)	0.50	0.51	0.53	0.55
Digestible proteins (%)	10.6	10.7	10.8	10.9
Digestible rabbit energy (kcal/kg)	2335	2374	2387	2412
Metabolizable rabbit energy (kcal/kg)	2187	2213	2222	2239
Cellulose VS ADF-ADL%	15.7	15.1	14.8	14.4
PD/ED calculated g/1000 kcal	48.4	48.6	48.6	48.7

DAKM=Detoxified apricot kernel meal, DTP=Dehydrated tomato pulp, CMV=Cytomegalovirus, NDF=Neutral detergent fiber, ADF=Acid detergent fiber, ADL=Acid detergent lignin

The animals were individually weighed at 33, 44, 58, and 77 days of age, and the feed was distributed at will; The consumption of each cage was checked every week of the experiment at a specific time. The average daily gains (ADG g/day), average daily intakes (ADI g/day), and conversion indices (CI) were calculated. The water was available *ad libitum* through the automatic pipette-type drinkers.

The slaughter parameters, carcass characteristics, and meat chemical composition were determined on the ten rabbits in each group according to the methods proposed by Dalle Zotte *et al*. [24].

### Slaughter parameters

Live weight at slaughter (LWS) (g); hot carcass weight (HCW) (g); cold carcass weight (CCW) (g); reference carcass weight (g); hot carcass yield (HCW/LWS×100); cold carcass yield (CCW/LWS×100); and muscle/bone ratio.

### Carcass characteristics

Liver weight (LW) (g); LW/live weight ratio at slaughter (LW/LWS×100); peri-renal fat weight (PRFW) (g); peri-renal fat/live weight ratio (PRFW/LWS×100); peri-renal fat/hot carcass ratio (PRFW/HCW×100); skin weight (SW)(g); SW/live weight ratio (SW/LWS×100); weight of digestive tract full (WDTF)(g); WDTF/LWS×100 ratio; front part weight (FPW)(g); rear part weight (RPW)(g); intermediate part weight “rable” (IPW) (g); FPW/HCW ratio (%); RPW/HCW ratio (%); and the ratio IPW/HCW (%).

### Meat quality

In the longissimus lumborum muscle, the pH was measured directly 24 h postmortem using a pH meter, and the chemical composition of meat was determined according to the AOAC method [21] with three replicates. The analyses included the water content, protein content, fat content, and mineral content. After 24 h in the cold room (6ºC), the weight of the cold carcass was determined.

### Statistical analysis

The data were prepared using the Microsoft Excel sheet. Statistical analysis and comparison of the means between the different diets (control and experimental) were performed by unidirectional analysis of variance using the SPSS software version 21 (IBM Corp., NY, USA). Student-Newman-Keuls and Duncan’s test were done if a significant difference at 5% standard error was found (p<0.05).

### Economic efficiency

The economic efficiency was calculated from the equation of Asar *et al*. [25]:

Economic efficiency (%) = [Net income/Total feed cost]×100.

Where:

Net income = Price of weight gain − Total feed cost.

Weight gain price = Average weight gain (kg/subject)×price of 1 kg body weight.

Total feed cost = Average consumption (kg /subject)×price of 1 kg of feed.

The cost of each kg of feed for the control and experimental diets was calculated based on the local market price of ingredients at the time of the experiment (the year 2018). Ancillary costs were not included.

- For the experimental batch, the total feed cost also included the cost of purchasing and detoxifying the apricot kernel cake and the cost of purchasing and dehydrating the tomato pulp (Table-3).
  The cost of the production of DAKM and DTP was calculated using the direct cost method, which consists of allocating the direct fixed costs specific to the production of apricot kernel cake. The common costs were not negligible, but they were reported on all the company’s products since they were difficult to evaluate in this study, which deals with only one byproduct.
- The basic wage in Algeria (SMIG) was 18,000 DZD/month if 35,000 DZD (gross wage+employer’s contributions) was assumed, since the average wage per month was about 200 DZD/h.
- The cost of energy was calculated by estimating the energy consumption of the material used for the production of oil cakes, knowing in the professional context, each kWh was charged at 4.472 DZD/kWh [26].
- To rinse one ton of oilcake with water, about 10 m^3^ of water was needed, at a price of 1 DZD/m^3^, according to the Algerian Water Agency [27].
- The depreciation of the equipment was estimated according to its purchase price (Dryer, Industrial Tank, etc.) on the market and according to the average capacity of the companies carrying out the oil extraction.

**Table- 3:** Data used to estimate the cost of DAKMs and DTPs.

Parameter	DAKM Value (DZD/T)	DTP Value (DZD/T)
Purchase	300	5
Transport cost	100	100
Truck unloading	35	35
Treatment		
The purchase of sodium bicarbonate	80	0
Cost of water for rinsing	1	0
The time required for treatment	1h/T	/
Cost per hour of work for treatment	172 DZD/h	/
Energy cost (drying)	9	10
Drying time	5 t/h	2 t/h
Total labor cost	175	10
Depreciation cost of equipment	200	100
Total	900	260

DAKM=Detoxified apricot kernel meal, DTP=Dehydrated tomato pulp

## Results

The substitution of soybean meal by DAKM and alfalfa hay by DTP had little effect on animal health status. Over the entire experimental period for all groups, a mortality rate of <6% was recorded.

### Zootechnical performances

The substitution of soybean meal with DAKM and dehydrated alfalfa with DTP improved the rabbit weights at 77 days for the experimental lots (Table-4). The weights at 44 days were higher for 40% and 60% groups. On day 58, the 30% group achieved better weight performance compared to the other groups (+65 points).

**Table-4:** Evolution of weight growth (g) and ADG (g/d) during fattening of bunnies as a function of the percentage of incorporation of the DAKM and DTP complex.

	0%	30%	40%	60%	SEM	p-value
Weight_(33d)_	808	811	810	814	8.66	0.7
Weight_(44d)_	1088^b^	1026^c^	1177^a^	1168^a^	11.51	0.01
Weight_(58d)_	1456^b^	1523^a^	1465^b^	1455^b^	13.25	0.03
Weight_(77d)_	1992^b^	2095^a^	2099^a^	2073^a^	16.86	0.01
ADG_(33-44d)_	25.45^b^	19.54^c^	33.36^a^	32.18^a^	0.99	0.04
ADG_(45-58d)_	28.30^b^	38.23^a^	22.15^c^	22.08^c^	0.88	0.03
ADG_(59-77d)_	29.77^c^	31.77^b^	35.22^a^	34.33^a^	1.84	0.01
ADG_(33-77d)_	28.19^b^	30.57^a^	30.69^a^	29.98^a^	0.94	0.03

ADG=Average daily gain (the indices indicate the period in days over which this parameter was calculated). The presence of different letters on the same line indicates a significant difference between diets (p < 0.05). DAKM=Detoxified apricot kernel meal, DTP=Dehydrated tomato pulp

The ADG (33-44 days) for the 40% and 60% groups was optimal and similar, with an increase of +8 points over the control group, with the 30% group having a minimum of −14 points over the experimental group, and −6 points over the control group. However, for the ADG (45-58 days), the 30% lot had a significantly higher value, with a difference of +16 points compared to the 40% and 60% lots, and −10 points compared to the control lot. For the experimental groups, the ADG (59-77 days) was significantly higher for the 40% and 60% lots, with the 30% lot having a lower value with −4 points. The control lot was the one with a lower value. Throughout the entire rearing period, the ADG (33-77 days) was significantly lower for the control lot (−2 points) compared to experimental lots with similar values.

The ADI and CI were similar (p>0.05) for all groups during the entire rearing period (33-77 days) (Table-5). The CI (33-44 days) for the 0% group was optimal (3.08) and remained similar for the other groups, with +0.23 points compared to the control group. The CI performance remained the same for all groups in the 44-58 days period. The CI (59-77 days) was depreciated and remained similar for the 0% and 30% groups, with a difference of +0.44 points compared with the 40% and 60% groups, which had similar values.

**Table-5:** Evolution of the CI (g/g) and ADI (g/days) during fattening of the young rabbits according to the percentage of incorporation of the DAKM and DTP complex.

Parameter	0%	30%	40%	60%	ESM	p-value
CI_(33-44d)_	3.08^b^	3.25^a^	3.36^a^	3.32^a^	0.65	0.01
CI_(45-58d)_	3.91	3.89	3.87	3.89	0.15	0.1
CI_(59-77d)_	4.22^a^	4.41^a^	3.92^b^	3.83^b^	0.47	0.03
CI_(33-77d)_	3.62	3.79	3.64	3.62	0.21	0.07
ADI_(33-44d)_	72.20^b^	77.24^a^	74.40^b^	73.51^ab^	1.31	0.02
ADI_(45-58d)_	93.60^b^	100^a^	95.63^b^	92.97^b^	2.01	0.04
ADI_(59-77d)_	117.20^a^	117.48^a^	114.74^b^	112.81^c^	1.15	0.02
ADI_(33-77d)_	96.00	98.26	94.92	93.10	4.1	0.14

On the same line, the averages with distinct letters are significantly different at the 5% threshold. CI=Consumption indexes, ADI=Average daily intakes, ADG=Average daily gain, DAKM=Detoxified apricot kernel meal, DTP=Dehydrated tomato pulp

The ADI (33-44 days) and ADI (45-58 days) were the highest (p>0.05) for the 30% group compared with the other groups. The ADI (59-77 days) for the 60% group was lower compared with the other groups, which had similar values.

### Slaughter parameters, carcass characteristics, and chemical composition of the meat

The incorporation of DAKM and DTP complex (up to 60%) as a substitute for soybean meal and alfalfa hay, respectively, significantly improved the slaughter parameters (p<0.05) (Table-6). The HCW and yields (HCW/LWS) from the 40% and 60% groups were similar and dominant over the 0% and 30% groups. The CCW of the experimental lots was higher (p<0.05) than that of the control group, and so was the yield (CCW/LWS), which was superior for the 40% and 60% groups; the muscle/bone ratio remained dominant for these groups.

**Table-6:** Evolution of slaughter parameters and carcass characteristics of young rabbits at fattening according to the percentage of incorporation of the complex apricot kernel cake and tomato pulp.

Slaughter parameters	0%	30%	40%	60%	ESM	p-value
LWS (g)	2138	2148	2128	2132	21.52	0.23
HCW (g)	1320^b^	1368^b^	1425^a^	1463^a^	7.09	0.01
CCW (g)	1273^b^	1340^a^	1387^a^	1425^a^	25.35	0.03
Yield HCW/LWS (%)	61.73^b^	63.71^b^	66.93^a^	68.62^a^	0.97	0.04
Yield CCW/LWS (%)	59.54^c^	62.40^b^	65.13^a^	66.85^a^	0.89	0.02
Yield muscle/bone	7.24^b^	7.86^b^	8.41^a^	8.03^a^	0.86	0.03
Carcass characteristics						
LW (g)	70^b^	74.3^a^	75^a^	75.6^a^	1.09	0.04
Ratio LW/LWS (%)	3.27^b^	3.46^a^	3.52^a^	3.55^a^	0.09	0.04
PRFW (g)	31.6^b^	34.6^a^	35^a^	36.6^a^	1.75	0.01
Ratio PRFW/LWS (%)	1,48^b^	1,61^a^	1,64^a^	1,72^a^	0,08	0.04
Ratio PRFW/CCW (%)	2.40^b^	2.53^a^	2.47^a^	2.51^a^	0.03	0.01
SW (g)	273.3^c^	281^b^	296.6^a^	293.3^a^	4.26	0.04
Ratio SW/LWS (%)	12.8^c^	13.1^b^	13.9^a^	13.8^a^	0.59	0.04
WFDT (g)	333.3	330.6	335	330	9.88	0.48
Ratio WFDT/LWS (%)	15.6	15.4	15.7	15.5	0.43	0.57
FPW (g)	233.3^b^	233.3^b^	284^a^	305^a^	16.24	0.02
RPW (g)	368.3^b^	385^a^	398^a^	408^a^	4.86	0.04
IPW (g)	286	287	281	290	6.12	0.64
Ratio FPW/HCW (%)	16.91^b^	17.06^b^	19.92^a^	19.82^a^	0.29	0.01
Ratio RPW/HCW (%)	27.90	28.18	28.05	27.89	0.93	0.62
Ratio IPW/HCC (%)	21.66	20.98	19.78	19.84	0.93	0.90
Chemical composition of the meat						
pH	6.04^b^	6.7^a^	6.77^a^	6.72^a^	0.04	0.01
Moisture content(% of DM)	65.43^b^	67.43^a^	67.28^a^	66.79^a^	0.34	0.01
Protein (% of DM)	20.55^b^	21.85^a^	22.07^a^	22.03^a^	0.23	0.01
Fat (% of DM)	7.7^b^	8.7^a^	8.87^a^	8.89^a^	0.26	0.01
Ash (% of DM)	1.02^b^	1.04^a^	1.05^a^	1.08^a^	0.03	0.01

In each line, the numbers followed by the same exponents do not differ significantly at p*<*0.05. LWS=Live weight at slaughter, HCW=Hot carcass weight, CCW=Cold carcass weight, LW=Liver weight, PRFW=Peri-renal fat weight, SW=Skin weight, WFDT=Weight of full digestive tract, FPW=Front part weight, RPW=Rear part weight, IPW=Intermediate part “rable” weight

The liver and PRFW of the experimental groups were higher than those of the control group (p>0.05), and so were the liver/Pva, Pgpr/Pva, and Pgpr/Pcc ratios. The SWs (pp) of the 40% and 60% lots were significantly higher (+14 points) than the 30% lot, which was +8 points higher than the control lot. The solid GI tract weights of all lots were similar (p<0.05). The weights of the front part of the control and 30% groups remained the same and lower than those of the 40% and 60% lots by about 50 points. The weight of the middle part remained unchanged for all groups. While similar, the weight of the rear part of the experimental groups was significantly higher than that of the control group.

The meat chemistry parameters of the experimental lots were significantly improved by substituting DAKM for soybean meal and DTP for alfalfa.

### Economic efficiency

The substitution of soybean meal with DAKM and alfalfa hay with DTP in the diet of growing rabbits had a positive effect on economic efficiency (Table-7). Indeed, the 60% group achieved optimal economic efficiency of 25.82% compared to the control group, and a 25.95% reduction in the total feed cost, saving 9.1 DZD for each kg of feed produced. As a result, the net income per kg of meat produced changes proportionally to the substitution rates of soybean meal with DAKM and alfalfa hay with DTP.

**Table-7:** Economic efficiency of replacing soybean meal with DAKM and alfalfa with DTP in fattening rabbits.

Parameters	0%	30%	40%	60%
Live weight at 33 days (g)	808	811	810	814
Live weight at 77 days (g)	1992	2095	2099	2073
Total weight gain (kg)	1.18	1.28	1.29	1.26
Price (DZD/kg live weight)	400	400	400	400
Incomes in total weight gain DZD/kg	473.60	513.60	515.60	503.60
Total feed intake/rabbit (kg)	4.03	4.13	3.99	3.91
Price of one kg of feed, DZD	38.43	33.91	32.39	29.35
The total cost of rabbit feed, DZD/kg	154.96	139.93	129.11	114.75
Economic efficiency (%)	3.06	3.67	3.99	4.39
Relative economic efficiency	100	120.10	130.67	143.60
Net income DZD/kg produced meat	318.64	373.67	386.49	388.85

DAKM = Detoxified apricot kernel meal, DTP = Dehydrated tomato pulp

## Discussion

The overall mortality rate was low (<6%). It was due to the transfer after weaning and the adaptation of the subjects to their new rearing conditions, as stated by De Blas [28]; however, it was still within the norms and range of what was recorded in national rabbit farms [29].

From the substitution of soybean meal with DAKM and alfalfa hay with DTP, the improvement of the overall performance of the experimental batches was mainly due to the increased efficiency in the use of experimental formulations without any change in feed intake level during the whole rearing period; it was probably dependent on the increase in the sulfur amino acid content of the experimental batches. Indeed, several authors, such as Colin *et al*. [30], Colin [31], Berchiche *et al*. [32], and Taboada *et al*. [33], agree that diets rich in sulfur amino acids allow better growth performance.

However, the study of Ahmed *et al*. [34] on the incorporation rates of 10%, 20%, and 30% DTP in the rabbit diet found no significant difference in growth performance compared to the control lot. The joint incorporation of DAKM and DTP resulted in an increase in crude cellulose and lignin, allowing the experimental batches a better valorization, as reported by Colin [31]; however, this remains dependent on the fiber source [35,36] and the nature of the parietal compounds [37]. The ADGs (33-77 days) in the white population of the experimental plots were similar to those reported by Hannachi-Rabia *et al*. [38] (29.3 g/days) and Lounaouci-Ouyed *et al*. [39] (30.4 g/days), lower than those reported by Benali *et al*. [40] (34 g/days) and Mennani *et al*. [10] (32 g/days), and higher than those of [41] (27.49 g/days) and [42] (23.80 g/days).

The CI values during the whole rearing period were similar to the results of [43] (3.2-3.6) and are within the standards recommended for intensive European rearing (3.60-3.82) [44].

All slaughter parameters were improved in proportion to the incorporation rate of DAKM and DTP (from 30% to 60%) (p<0.05), except for the weight of the full digestive tract and saddle. However, for DAKM and DTP incorporation rates of 10-30%, Mennani *et al*. [9], Mennani *et al*. [10] reported no differences in slaughter parameters and carcass characteristics between the batches. This suggests that these agro-industrial byproducts, especially DAKMs, incorporated in up to 60% in rabbit fattening diets, did not affect the digestive tract and saddle weights.

The nutritional value of rabbit meat is highly variable [45,46], resulting in a highly variable chemical composition depending on the part of the carcass studied [47] and different production factors [48], especially the feed [49]. In this sense, the introduction of DAKMs and DTPs in rabbit fattening diets induced better meat composition performances in the experimental lots (30%, 40%, and 60%) compared to the control lot, while remaining similar between them. For lower levels of DAKM (10%, 20%, and 30%), Mennani *et al*. [9], Mennani *et al*. [10] observed an improving meat chemical composition; this remained different between the experimental lots, with a significant decrease in fat content which, on the other hand, significantly increased in our study. This was contrary to the increase in the fat content of experimental lots due to higher DAKM incorporation rates.

The price per kilogram of feed produced by the experimental 60% group created a financial gain of 40.21 DZD due to the low-cost price of agro-industrial byproducts and inflationary prices of soybean meal and alfalfa, which are entirely imported, depending on the fluctuations in world stock markets.

## Conclusion

The DAKM/DTP complex can be considered a good source of protein (58.41%) and fiber (45.62%). This can be used as an alternative to the soybean cake/alfalfa hay complex, with a substitution rate of up to 60%, without any negative effects on growth rates, food conversion, and food consumption. It improved the live weights, all slaughter parameters, carcass characteristics, and meat chemical composition while reducing the cost of feed consumed. These encouraging results allow us to put forward the idea of increasing the substitution rates to determine the optimal incorporation rates.

## Authors’ Contributions

LO: Prepared the ground conditions and collected the data. YA: Performed the analysis of the data. AM: Carried out and drafted the economic analysis. FA: Designed the study and drafted it. RA: Revised the manuscript. All authors have read and approved the final manuscript.
